# High-performance electrochemical sensors based on doped C_60_ fullerene for non-invasive diabetes diagnosis and environmental acetone removal: a computational study

**DOI:** 10.1038/s41598-025-24911-x

**Published:** 2025-11-20

**Authors:** Mohammed Ghazwani, Umme Hani

**Affiliations:** Department of Pharmaceutics, College of Pharmacy, King Khalid University, Al Faraa, Abha, 62223 Saudi Arabia

**Keywords:** Biosensor design, Environmental pollutant, Acetone detection, Type 2 diabetes, Electrochemical sensor, Chemistry, Environmental sciences, Materials science, Nanoscience and technology

## Abstract

This study develops chemical sensor platforms using Zn- and Al-doped C_60_ fullerenes to sense acetone (a significant biomarker for type 2 diabetes and a prevalent environmental pollutant). A comprehensive suite of DFT and QTAIM calculations was carried out to systematically investigate the structural, electronic, and sensing characteristics of the interaction of acetone with a pristine (C_60_) fullerene as well as Al-doped (AlC_59_) and Zn-doped (ZnC_59_) fullerenes. These studies consisted of MEP mapping, frontier orbital (HOMO-LUMO) energy analyses, Natural Bond Orbital (NBO), and Non-Covalent Interaction (NCI) analyses. According to this data, doping does indeed greatly improve sensor performance. In particular, the ZnC59 sensor exhibited remarkable properties: the lowest energy gap of 0.31 eV, a high electrical conductance of 7.40 × 10^6^ A.m^− 2^, a high charge transfer (ΔNmax = 28.0), and a fast recovery time of 2.51 × 10^− 8^ s. The AlC_59_ was more efficient for acetone detection with the strongest adsorptive ability (-50.2 kcal.mol^− 1^) as an exceptional adsorbent. The ZnC_59_ complex exhibited the most advantageous characteristics, including strong but reversible binding to acetone, high sensitivity, and rapid regenerability. The interaction in ZnC_59_@Ac was favorable and of intermediate strength, as determined by QTAIM and NCI analyses. Overall, this research aims to validate Zn-doped C_60_ for use in sensitive, reusable electrochemical sensing devices for detecting breath acetone. At the same time, Al-doped C_60_ shows promise for environmental acetone adsorption.

## Introduction

Diabetes is a chronic metabolic disorder with high blood sugar levels due to a lack of insulin or a lack of effective utilization of insulin, which is a hormone that regulates glucose levels. Diabetes is usually split into two types: Type 1 diabetes, an autoimmune disorder where the pancreas does not produce insulin or produces very little; and type 2 diabetes, which is more common and generally associated with lifestyle factors like obesity, poor diet, and lack of exercise. Gestational diabetes can occur during pregnancy, and those who have it are at an increased risk of developing type two diabetes in the future. It is important to detect diabetes early and proactively screen for diabetes, as diabetes may be asymptomatic at early stages of the disease, particularly Type 2 diabetes. If left untreated, diabetes may lead to significant morbidity including cardiovascular disease, damage to the kidneys, damage to nerves, and possible loss of vision^1,2^. Screening allows us to detect people who are at risk for or early stages of diabetes so that we can intervene with lifestyle modification, medical treatment, and monitoring to prevent or delay complications to improve health outcomes and quality of life. There are common methods of diagnosis, including: fasting blood glucose (FBG), oral glucose tolerance test (OGTT), random blood glucose test, and glycated hemoglobin (HbA1c) test^3^.

FBG testing indicates blood sugars following an overnight fast, and it is the standard for diagnosing diabetes and prediabetes. OGTT assesses how the body responds to glucose after consuming a sugary drink, and it is commonly used for diagnosing gestational diabetes. Random blood glucose tests provide blood glucose values anytime of the day and do not require before fasting before testing. HbA1c is testing reflects the average blood sugars for a two-to-three-month period and is valuable for longer-term trend monitoring^4^. These are all useful, but they have useful and meaningful limitations. Many require trained operators, rely on costly and sophisticated instruments, require invasive blood sampling, or simply will not be available in low-resource settings. And with it, the need for a more user-friendly, available, and reliable method to enable early diagnosis and greater screening of diabetes will only increase.

Electrochemical sensors are becoming useful devices to detect early disease states by measuring biomarkers in exhaled air. Many sensors have been developed to detect VOCs related to different diseases, providing a unique way forward with the idea of non-invasive disease diagnostic or identification methods^5^. Zhou et al. have proposed that acetone (Ac) present in exhaled air, could serve as a biomarker for non-invasive diagnosis of type 2 diabetes mellitus through elevations in acetone levels that are suggestive of glucose metabolism disturbance^6^. The literature has also supported acetone as an environmental pollutant and a significant contributor to overall air pollution with exposure risks for humans or ecosystems^7^. Acetone is a VOC that does as well as being introduced into the environment by industry, the use of industrial solvents, and waste emissions. Acetone is highly volatile as a source of VOC, meaning it escapes relatively quickly into the environment. Once added to the atmosphere acetone can photochemically react and create ground level ozone or secondary organic aerosols (two pollutants related to causing smog or respiratory issues). Research like that of Khader et al. has stressed that acetone concentrations in both industrial wastewater and the air in and around buildings need to be monitored and regulated due to their persistence in the environment and their participation in atmospheric processes that degrade urban air quality^8^. These studies highlight the need for materials that can efficiently not only detect acetone in air and wastewater but also sequester or remove it for environmental remediation.

Thus, given that acetone is both a biomarker of diabetes and an environmental pollutant, it is important to build rapid and efficient detection methods for acetone. Acetone detection using electrochemical sensors is important for two reasons; in environmental applications, it is used for pollution mitigation as well as aiding in revitalization, and in a biomedical context it is to quickly diagnose and detect diabetes so that timely intervention can occur and allow for effective disease management. These diagnostic electrochemical sensors that were designed and synthesized based on using nanoparticles have several advantages. They are non-invasive, always available, user-friendly, no special personnel or expensive parts or equipment are required, and most importantly, it leads to broader accessibility, especially in the proud of-cereal tests and settings of lower resource. Numerous nanoparticles have been utilized to design electrochemical sensors for the purpose of enhance either the: sensitive, selectivity, stability, and performance^9,10^. Examples of nanoparticles that are being developed for electrochemical sensors usable in fields of both health and environmental applications include carbon nanotubes, fullerenes, and carbon nanosheets such as graphene and graphene oxide (and fullerenes especially fullerene C60) have generated much sovereign interest due to their mechanical- and electrical- unique properties.

Composed of 60 carbon atoms in a spherical geometry resembling a soccer ball, fullerene C60 presents a number of advantages over other nanomaterials. The first is due to its symmetric three-dimensional structure, which possesses a relatively high surface area that may serve as an efficient interface with Dads’s target biomolecules such as acetone to rapidly enhance the performance of a sensor. Further, fullerene C60 displays excellent electron-accepting properties, improving the overall rate of electron transfer in electrochemical sensing leading to increases in sensitivity. Lastly, fullerene C60 possesses chemical stabilities, low toxicity, and excellent antioxidant properties that further enhance its applicability in biomedical use. Overall, fullerene C60 represents a great candidate to enhance sensors in association with biomarkers present in exhaled air^11^.

Recent studies have noted the increasing interest in fullerene C60 as a nanomaterial for developing diagnostic sensors because of its unique properties such as high electron mobility, large surface area and excellent chemical stability, making it a readily attractive nanomaterial for detecting biomarkers and analytes. While the potential use of fullerene C60 as a nanomaterial in the development of sensors has been explored in different ways in some literature, there have been a number of significant developments, and the following studies added profound effects in the application of fullerene C60 for sensitization^12,13^. In one study, Sutradhar et al. demonstrated the use of a new composite nanomaterial with fullerene-C60-nanogold for non-enzymatic glucose detection. In this novel composite, the authors utilized the unique electronic properties of fullerene C60 combined with the catalytic properties of nanogold to achieve high selectivity and sensitivity in glucose sensing, both of which are essential when monitoring diabetes^14^. The other study by Bashiri et al. used fullerene C60 nanomaterial for rapid electrochemical detection of amphetamine, which is a psychoactive drug. This work illustrated the ability of fullerene C60 to develop a rapid, simple and accurate sensor in the area of drug screening^15^. However, Hadi et al. used functionalized C60 nanocages for detection of methamphetamine. In this study, the use of chemically functionalized fullerene C60 had a greater interaction with the target molecule, an important feature that resulted in increased sensitivity and rapid detection, and also demonstrated a potential for use as an adsorbent and sensor^16^.

The purpose of this study is to expand on MG Zhou’s findings related to the safe use of acetone as a biomarker for non-invasive diagnosis of type 2 diabetes and the capabilities of fullerene C60 to act as an electrochemical sensor. An innovative acetone sensor based on fullerene C60 will be designed and discussed in this article that could serve as an appropriate non-invasive diagnostic tool for early diagnosis of type 2 diabetes. Doping may alter the electronic structure of fullerene C60 to favor acetone over other molecules. Therefore, we also analyze the sensing properties of the initial C60 fullerene doped forms with aluminum (Al) and zinc (Zn). The use of Al, an electron-deficient atom, improves the electrophilic interactions occurring between fullerene and acetone and could improve sensitivity^17^. As for zinc, due to its moderate electronegativity and catalytic properties, it may increase charge transfer and adsorption of acetone molecules, which would be beneficial to sensor performance^18^. Of course, we acknowledge that this is not the end of the road, and doping with other atoms could be considered a valuable next step.

In this work, we utilized density functional theory (DFT) calculations along with the quantum theory of atoms in molecules (QTAIM)^19,20^. Both of these theoretical methods provide a thorough understanding of the interaction mechanisms of acetone with both pristine and doped fullerene C60 and permit the determination of adsorption energies, charge transfer properties, and sensor selectivity. The goal of this work is to contribute towards a new class of sensitive electrochemical sensors and effective adsorbents for acetone detection. This would enable rapid, non-invasive type 2 diabetes diagnosis, while also providing an effective means to remove acetone as an environmental contaminant.

## Computational method

**Fig. 1:**
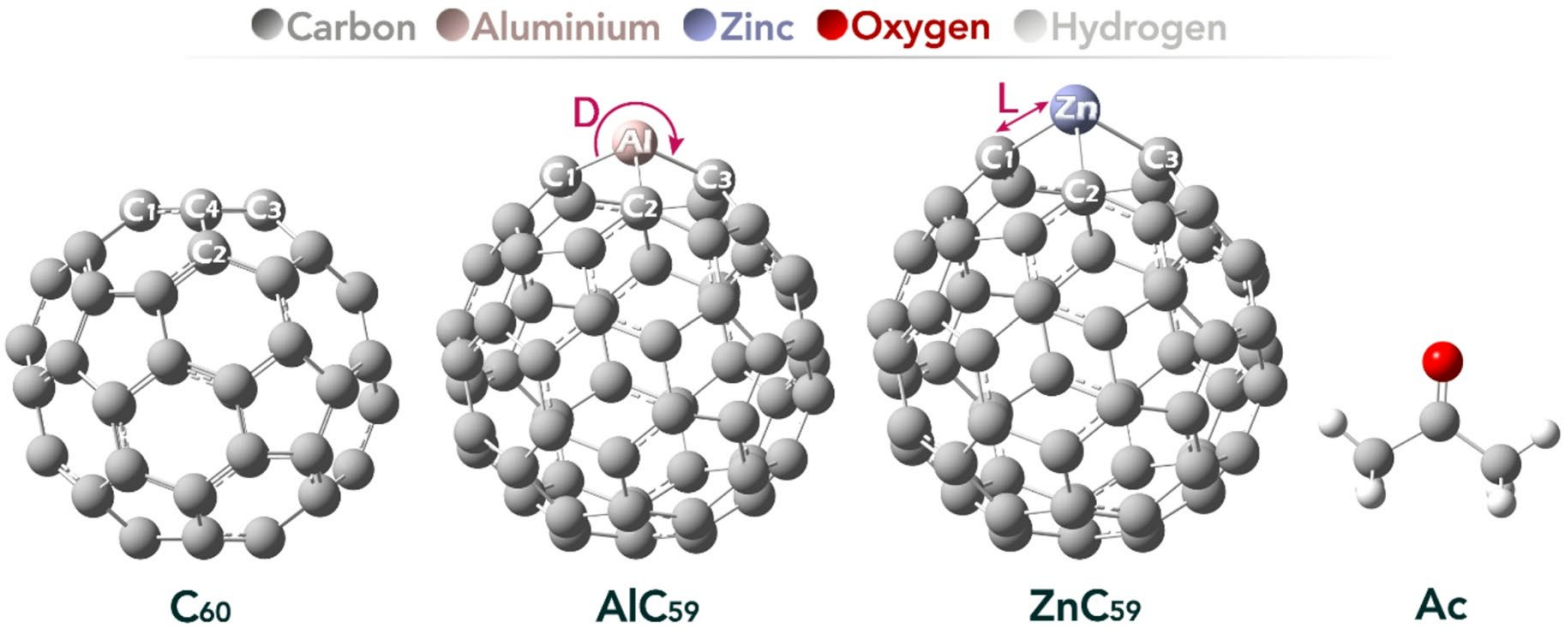
Optimized geometry of each of the structures designed in this work.

In this study, the molecular structures of pristine C60 fullerene, its Zn and Al doped forms and their complexes with acetone were initially built by GaussView 6.0 software, subsequently geometry optimization were carried out with Gaussian 09 W (Fig. 1)^21^. All geometries were optimized at the DFT/B97D/6-31G* level of theory in aqueous phase using the Conductor-like Polarizable Continuum Model (CPCM)^22,23^. Spin multiplicities were carefully checked for all systems. Zinc, with its d10 configuration, remains a closed-shell system and was therefore treated consistently with a singlet state. For aluminum doping, which could in principle lead to open-shell behavior due to its 3s²3p¹ configuration, we explicitly performed spin checks using both restricted and unrestricted DFT formalisms at the B97D/6-31G* level. In all cases, the restricted singlet solutions were stable and no spin contamination was detected. The vibrational frequency analyses confirmed true minima without imaginary frequencies, further verifying that the optimized geometries correspond to ground-state singlets. The B97D functional was chosen because it incorporated the empirical dispersion corrections which allow accurate description of all the non-covalent interactions such as π-π stacking and van der Waals interactions (which are key interactions to establish the sensing performance of fullerene-based systems)^24^. It is also relevant to mention that the B97D functional has been reported as a suitable theoretical method to calculate HOMO-LUMO energy gap of C60 fullerene, with results generally matching experimental data well^25^.

The cohesive energy (E_Coh_) for each of the designed systems was calculated using Eq. 1 to assess their structural stability.

1 $$\:{E}_{Coh}=-({E}_{Complex}-\sum\:{E}_{\text{i}\text{n}\text{d}\text{i}\text{v}\text{i}\text{d}\text{u}\text{a}\text{l}})/n$$

To calculate E_Coh_, the difference between the complex energy (E_Complex_) and the sum of the total atomic energies is divided by the total number of atoms in the system ()^26^. To ensure an unbiased comparison across systems with different atomic compositions (pristine C60, AlC59, and ZnC59), the cohesive energy was normalized per atom by dividing it by the total number of atoms (n) in each system. Since all systems have the same total number of atoms (*n* = 60), the normalization is constant across all structures, and the coherence energy, independent of chemical composition or doping, represents the average stability per atom in each system. Thus, the comparison is not affected by system size or stoichiometry, but rather reveals the intrinsic stability provided by the dopant in the carbon framework of the same size.

The electronic properties of the designed systems (including energy gap (HLG), chemical softness (S), chemical hardness (η), and chemical potential (µ)) were calculated to gain deeper insight into their chemical reactivity and sensing behavior. These parameters were determined using the energies of the highest occupied molecular orbital (HOMO) and the lowest unoccupied molecular orbital (LUMO), according to Eqs. 2–5^27^:

2 $$\:\text{H}\text{L}\text{G}=\left|{\text{E}}_{\text{H}\text{O}\text{M}\text{O}}-{\text{E}}_{\text{L}\text{U}\text{M}\text{O}}\right|$$

3 $$\:{\upeta\:}=\raisebox{1ex}{$(-{\text{E}}_{\text{H}\text{O}\text{M}\text{O}}-(-{\text{E}}_{\text{L}\text{U}\text{M}\text{O}}\:\left)\right)$}\!\left/\:\!\raisebox{-1ex}{$2$}\right.$$

4 $$\:{\upmu\:}=-(-{\text{E}}_{\text{H}\text{O}\text{M}\text{O}}+(-{\text{E}}_{\text{L}\text{U}\text{M}\text{O}}\left)\right)/2$$

5 $$\:S=1/2{\upeta\:}$$

To further investigate the charge transfer characteristics of the designed systems, two important descriptors were calculated: the maximum charge transfer (ΔNmax) and the electrophilicity-based charge transfer (ECT)^28^. These parameters provide insight into the donor-acceptor behavior of the sensor-analyte complexes and help quantify the extent and direction of charge transfer. They were calculated using Eqs. 6 and 7:

6 $$\:{\varDelta\:N}_{max}=-\raisebox{1ex}{$\mu\:$}\!\left/\:\!\raisebox{-1ex}{$\eta\:$}\right.$$

7 $$\:ECT={\left({{\Delta\:}N}_{max}\right)}_{\alpha\:}-{\left({{\Delta\:}N}_{max}\right)}_{\beta\:}$$

In this equation, (ΔNmax)α is the maximum charge transfer value of species α (C_60_), and (ΔNmax)β is the maximum charge transfer value of species β (Ac). Suppose ECT > 0, C60 (α) has a greater ability to accept electrons than Ac (β). Charge transfer occurs from acetone to C60. If ECT < 0, Ac (β) has a greater ability to accept electrons than C60 (α). Charge transfer occurs from C60 to acetone^29^.

The adsorption energy of acetone on C_60_ fullerene and its doped forms was calculated using Eq. (8), which quantifies the strength and stability of the interaction between acetone and the sensor surface.

8 $$\:{E}_{ads}={E}_{Complex}-\left({E}_{\text{A}\text{c}}+{E}_{\left(R-\right)C60}\right)+{E}_{BSSE}$$

where, E_ads_: is the adsorption energy, E_Complex_: is the total energy of the optimized acetone-fullerene (or doped fullerene) complex, E_R−(C60)_: is the energy of the isolated fullerene or doped fullerene, E_Ac_: is the energy of the isolated acetone molecule^30,31^. Also, the calculated adsorption energies (Eads) include a correction for the Basis Set Superposition Error (BSSE) using the standard counterpoise method proposed by Boys and Bernardi^32^.

The recovery time ($\:\tau\:$) and electrical conductivity ($\:{\upsigma\:}$) of the designed structures were calculated using Eqs. (9) and (10), respectively, to evaluate their effectiveness as sensors.

9 $$\:\tau\:={V}_{0}^{-1}\times\:\text{e}\text{x}\text{p}(-\frac{{E}_{ads}}{{k}_{B}T})$$

10 $$\:{\upsigma\:}=A{T}^{3/2}{e}^{(-HLG/2KT)}$$

In these equations: A = Richardson constant (6 × 10^5^ A.m^− 2^), k_B_= Boltzmann constant, E_ads_= adsorption energy, V_0_ = attempt frequency (10^12^ s^− 1^), and, T = temperature (298 K)^33^.

These calculations help assess the practical sensing performance of each designed structure by combining stability (through recovery time) and sensitivity (through conductivity changes). These computational insights provide a robust foundation for understanding the sensing mechanisms of the designed materials and offer valuable guidance for future experimental development of efficient and selective sensors.

## Results and discussion

### Structural properties

#### Bond length and bond angle

The investigation of bond lengths (L) and bond angles (D) is critical to the design of electrochemical sensors because the bond lengths and angles are directly related to the electronic properties and interaction sites of the sensor material. Doping alters the local atomic environment, resulting in modified bond lengths and angles, which influences the π-bonding framework. Modifications in π bonding also alter electron delocalization and orbital overlap, which is critical to efficient charge transfer between the sensor and analyte. Tracking these geometric changes will help illustrate how doping affects the sensor’s sensitivity and selectivity by modifying its electronic structure to facilitate binding interactions with the analyte^34^.

**Table 1:** Bond length (in Angstroms (A)) and bond angle (in degrees (^o^)) values in each of the sensors designed in this work.

Structure	L (A)		D (^o^)	
C_60_	C_1_-C_4_	1.40	C_1_-C_4_-C_3_	120.00
	C_2_-C_4_	1.45	C_1_-C_4_-C_2_	119.99
	C_3_-C_4_	1.45	C_2_-C_4_-C_3_	108.00
AlC_59_	C_1_-Al	1.90	C_1_-Al-C_3_	104.04
	C_2_-Al	1.92	C_1_-Al-C_2_	104.04
	C_3_-Al	1.92	C_2_-Al-C_3_	92.19
ZnC_59_	C_1_-Zn	1.97	C_1_-Zn-C_3_	99.55
	C_2_-Zn	2.03	C_1_-Zn-C_2_	91.95
	C_3_-Zn	2.02	C_2_-Zn-C_3_	82.00

A comparison of the bond lengths and bond angles in Table 1 reveals notable structural changes in the fullerene framework upon doping with Al and Zn. In pristine C_60_, the bond lengths (C-C) range from 1.40 to 1.45 Å, and the bond angles are nearly ideal for sp^2^ hybridization, ranging from 108.00° to 120.00°, reflecting the symmetric and delocalized π-bonding characteristic of the undoped fullerene.

Upon doping with aluminum (AlC_59_), the carbon-carbon bonds are replaced with Al-C bonds, which show a significant increase in bond length to 1.90–1.92 Å. The bond angles also decrease, particularly the C_2_-Al-C_3_ angle, which drops to 92.19°, indicating a notable distortion in the local geometry due to the larger atomic radius and different bonding characteristics of aluminum compared to carbon. These changes suggest a disruption in the delocalized π system and localized electron density around the doping site.

In the case of zinc doping (ZnC_59_), an even greater increase in bond lengths is observed, ranging from 1.97 to 2.03 Å. The bond angles are further reduced, with the smallest angle (C_2_-Zn-C_3_) reaching 82.00°, indicating a more pronounced structural distortion compared to Al doping. This reflects zinc’s limited ability to engage in π bonding, leading to stronger localization of electron density and a significant alteration of the fullerene’s electronic structure.

Overall, both Al and Zn doping introduce notable changes in bond lengths and angles, with Zn causing more severe distortion. These geometric modifications influence the electronic distribution and may enhance the sensor’s reactivity and charge transfer properties by modifying the active site geometry and local electronic environment.

#### Cohesive energy

**Fig. 2:**
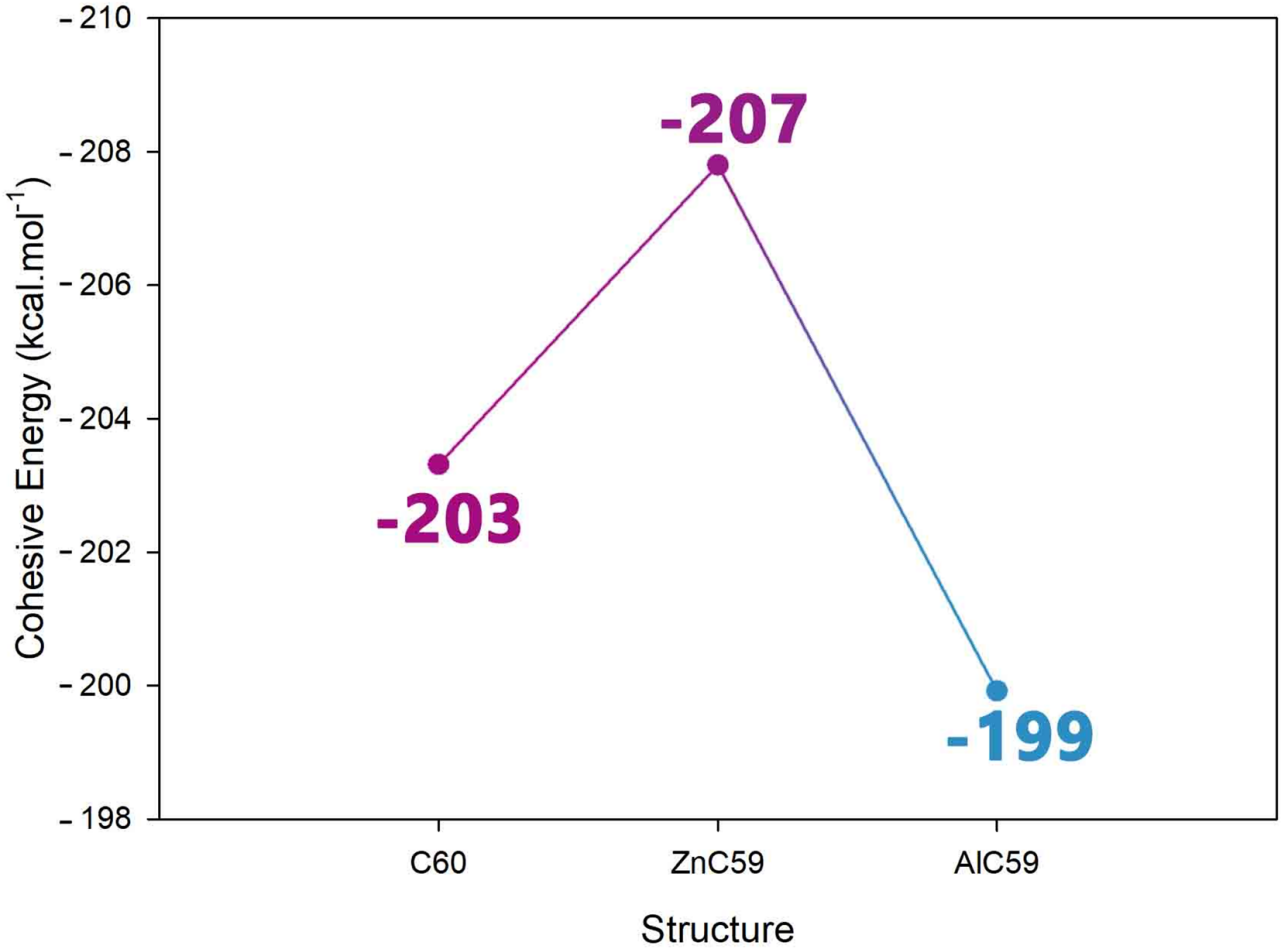
Cohesive energy changes after doping C_60_ with Al and Zn.

Cohesive energy can be understood as the amount of energy necessary to separate a compound into its isolated atoms, and it serves as an overall measure of the stability and bonding strength within a structure. The assessment of cohesive energy is important in the creation of electrochemical sensors, as it can provide insight into the structural resiliency and durability of the sensor material under an applied operational potential. A more negative cohesive energy signifies a more stable and energetically favorable structure, which is important if you want the sensor to perform degeneration or aging free as time develops. In this work, we calculated the cohesive energy of the pristine C60 fullerene, as well as its aluminum and zinc doped forms, for comparison relative to their structural stability^35^. The calculations appear in Fig. 2.

Cohesive energy data for C60, ZnC59, and AlC59 reveal the role of doping in the stability of the designed structures. The cohesive energy of C60 (−203 kcal/mol) is reflective of its high stability due to the symmetry of the delocalized π-electron system characteristic of the fullerene. Substituting a carbon atom with a zinc atom raises the cohesive energy to −207 kcal/mol, indicating that the zinc dopant increases the stability of the structure. This increase is likely a result of favorable zinc-carbon bonding interactions and some minimal disruption of the electronic characteristics, indicating that the zinc dopant is stable within the carbon framework. The cohesive energy of the aluminum compound is reduced from −203 kcal/mol to −199 kcal/mol, suggesting that the aluminum atom slightly destabilized the structure. The reduced stability seems to show that the incorporation of aluminum atoms provides less favorable interactions with the surrounding carbon atoms, which may reflect atomic size interaction, valency differences, or less effective atomic overlap. The structural strain, atomic size imbalance and/or orbital interaction shows that the amine ends of the aluminum compound possess weaker bonds and are less cohesive than the carbon cage. The results suggest that depending on the nature of the dopant, fullerene structures may become more stable or destabilized by doping, which may result in valuable alternative structural properties. The increase in cohesive energy with Zn indicates a stabilizing effect, while the decrease with Al reflects a modest destabilizing influence.

Beyond theoretical stability, it is important to consider the experimental feasibility of substitutional doping in C60. Previous reports confirm that heteroatom-doped fullerenes can indeed be synthesized, either through high-energy methods such as arc-discharge and laser ablation, or via metallofullerene precursors. In particular, substitutional doping with main-group and transition metals has been achieved experimentally, demonstrating that such modifications are accessible in practice^36–38^. Our cohesive energy calculations further support this feasibility, showing that Zn substitution enhances the intrinsic stability of the carbon framework, while Al substitution introduces only a modest destabilization. These findings align with experimental observations that certain dopants incorporate more favorably than others, validating the use of AlC59 and ZnC59 as realistic models for exploring fullerene-based sensors.

### Molecular electrostatic potential (MEP) map

**Fig. 3:**
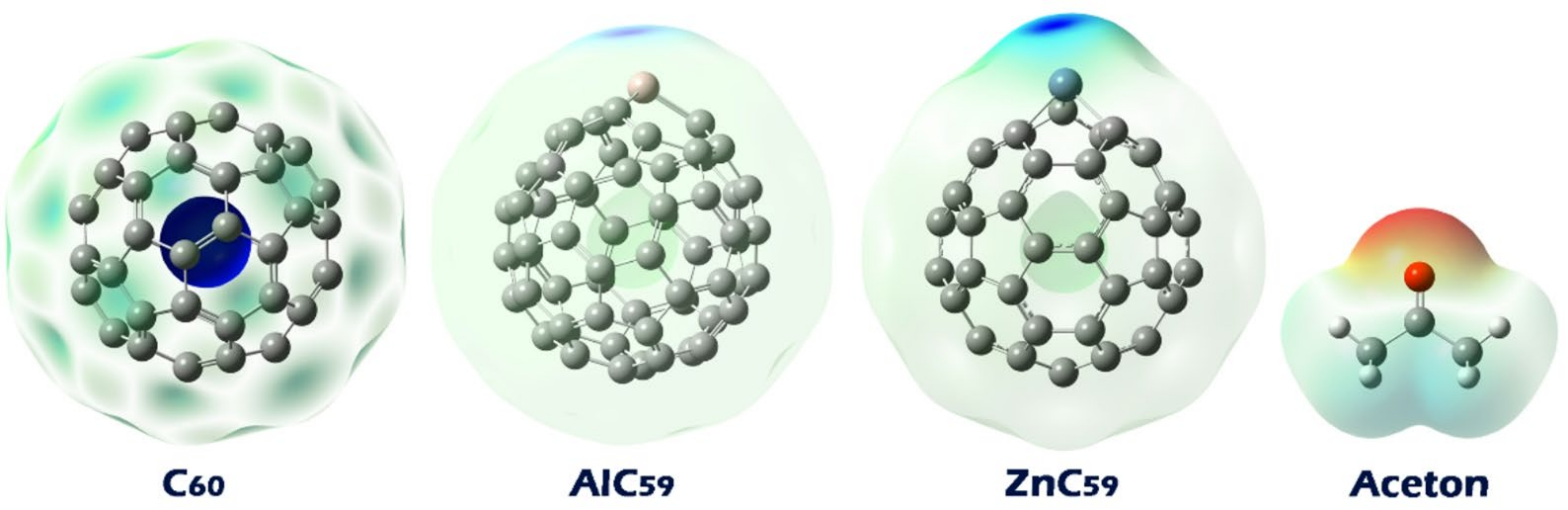
MEP contours in each of the structures designed in this work.

Examining the Molecular Electrostatic Potential (MEP) map is crucial for identifying the most likely sites of interaction between molecules, as it visually represents the distribution of electrostatic potential across the molecular surface. This distribution indicates regions of electron density accumulation and depletion, which correspond to potential nucleophilic and electrophilic sites, respectively. Understanding these regions helps predict where intermolecular interactions such as hydrogen bonding, electrostatic attractions, or nucleophilic attacks are most likely to occur, providing valuable insight into molecular recognition and binding mechanisms^39^. In the MEP contour, colors are used to represent different electrostatic potential values: red indicates regions of high electron density with negative potential, often associated with nucleophilic sites; blue represents areas of low electron density with positive potential, corresponding to electrophilic sites; and green denotes regions of neutral or intermediate potential. Analysis of these colored regions enables the investigation of reactive sites on a molecule to understand its interaction behavior^40^. For this purpose, MEP maps for acetone, pristine C60, and its doped forms were reported in Fig. 3.

**Fig. 4:**
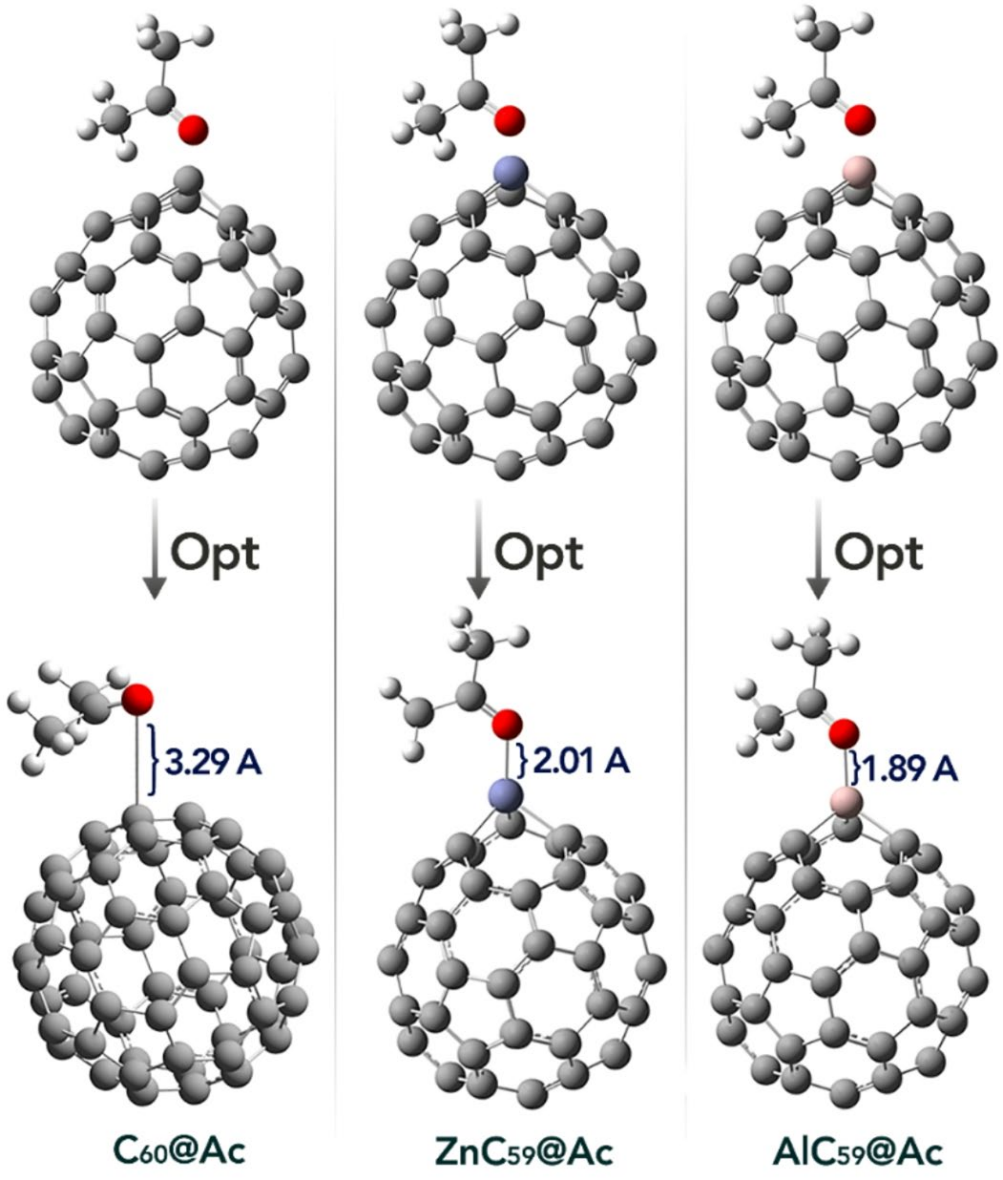
Optimized geometry of each of the designed complexes.

Based on the described Molecular Electrostatic Potential (MEP) maps, the most likely interaction points between acetone and pristine or doped C_60_ can be identified. In pristine C_60_, the blue color is uniformly distributed around the molecule, indicating a generally positive electrostatic potential evenly spread over the surface. This suggests that electrophilic sites are delocalized and no specific localized interaction points dominate, meaning acetone may interact more broadly but less specifically with the fullerene surface. In contrast, for the doped fullerenes AlC_60_ and ZnC_60_, the blue color is concentrated around the dopant atoms, indicating that these atoms possess a higher positive electrostatic potential and act as distinct electrophilic centers. The green color spread across the rest of the molecule indicates regions of neutral potential. Meanwhile, acetone’s MEP map shows a red region localized on the oxygen atom, highlighting it as a site of high electron density and nucleophilicity. Therefore, the oxygen atom in acetone is most likely to interact with the Al and Zn atoms in AlC_59_ and ZnC_59_. Based on these results, each of the desired complexes was designed, and the optimal geometry of each of them is shown in Fig. 4.

The bond lengths between the oxygen atom of acetone and the sensor surface in the studied complexes show clear variation that reflects differences in molecular interactions and potential electronic communication. In the pristine C_60_@Ac complex, the O-C bond length of 3.29 Å indicates a relatively large spatial separation between the oxygen atom of acetone and the fullerene surface. This longer distance suggests limited orbital overlap, which can restrict efficient π-electron transfer between acetone and the sensor.

In contrast, the doped complexes ZnC_59_@Ac and AlC_59_@Ac exhibit significantly shorter bond lengths of 2.01 Å and 1.89 Å, respectively, between the oxygen atom and the dopant metal centers. These reduced distances imply a stronger spatial interaction and more effective approach of the acetone molecule toward the dopant sites. Such proximity facilitates enhanced orbital overlap between the lone pair electrons of oxygen and the vacant orbitals or partially filled d-orbitals of the dopant atoms. This increased overlap is critical for promoting π-electron delocalization and charge transfer processes, which are fundamental to the sensing mechanism in fullerene-based materials.

Furthermore, the difference in bond lengths between the Zn-O and Al-O interactions may relate to the atomic size and electronic configuration of the dopant atoms, influencing their coordination environment and electron-accepting ability. The slightly shorter Al-O bond length indicates a closer interaction, which could potentially lead to more effective electron communication at the interface.

### Energy of HOMO/LUMO frontier orbitals and quantum parameters

In electrochemical sensor design, parameters like the HOMO-LUMO energy gap (HLG), chemical hardness (η), softness (S), chemical potential (µ), maximum charge transfer (∆Nmax), and electrophilicity-based charge transfer (ECT) are essential for understanding sensor behavior. HLG indicates electronic excitation and charge transfer ability, while hardness and softness reflect reactivity and stability. Chemical potential influences electron donation or acceptance. ∆Nmax measures the potential extent of electron transfer, and ECT reveals the direction and magnitude of charge flow. Together, these factors help predict and optimize sensor performance by clarifying how the sensor interacts electronically with target molecules^41^. Each of these parameters was calculated and the results were reported in Table 2.

**Table 2:** Energy values of the HOMO/LUMO orbitals, HLG, η, S, µ, ∆Nmax, and ECT for each of the designed sensors in the presence and absence of adderall (all units are in eV and only S is in eV^− 1^).

Structure	LUMO	HOMO	HLG	η	S	µ	∆Nmax	ECT
Ac	− 1.21	− 5.31	4.1	2.05	0.24	− 3.26	1.59	–
C_60_	− 3.58	− 5.25	1.67	0.83	0.59	− 4.41	5.28	–
AlC_59_	− 4.26	− 5.06	0.80	0.40	1.25	− 4.66	11.65	–
ZnC_59_	− 4.09	− 4.45	0.36	0.18	2.77	− 4.27	23.72	–
C_60_@Ac	− 3.78	− 4.43	0.65	0.32	1.53	− 4.10	12.81	− 7.53
AlC_59_@Ac	− 4.18	− 4.93	0.75	0.37	1.33	− 4.55	12.31	− 0.66
ZnC_59_@Ac	− 4.05	− 4.36	0.31	0.15	3.22	− 4.20	28.0	− 4.28

To comprehensively evaluate the sensing performance of the designed fullerene-based structures for acetone detection, we analyze the data presented in Table 2 in two main steps: first, the effect of doping on the pristine C_60_ fullerene is examined, and second, the impact of acetone complexation on each doped and undoped sensor is assessed.

Initially, it is important to highlight that the calculated energy gap (HLG) for pristine C_60_ is 1.67 eV, which closely matches the experimental value reported by T. Rabenau et al.^42^. This agreement confirms the accuracy and reliability of the computational methodology used in this work, further validating the theoretical predictions for the other sensor designs.

Doping the C_60_ structure with metal atoms (Al and Zn) significantly alters its electronic properties. For AlC_59_, the energy gap decreases drastically to 0.80 eV, indicating increased reactivity and reduced kinetic stability. This trend is even more pronounced in ZnC_59_, which exhibits an exceptionally low energy gap of 0.36 eV. These reductions in HLG are accompanied by increases in global softness (S) and maximum charge transfer (∆Nmax), particularly in ZnC_59_ (∆Nmax = 23.72), highlighting a greater potential for electron donation or acceptance, which is crucial for sensing applications.

Upon complexation with acetone, all structures show further reductions in energy gap, indicating strong electronic interaction between the sensor and the analyte. In C_60_@Ac, the HLG drops from 1.67 to 0.65 eV, while AlC_59_@Ac shows a modest change from 0.80 to 0.75 eV. The most significant change is observed in ZnC_59_@Ac, where the HLG decreases from 0.36 to just 0.31 eV. This consistent reduction in the energy gap upon acetone binding suggests successful complex formation and a detectable change in electronic properties, which is desirable in a chemical sensor.

The change in electronegativity (µ) and chemical hardness (η) also support this trend. The decrease in η upon acetone complexation, especially in ZnC_59_@Ac (η = 0.15 eV), along with the highest global softness (S = 3.22 eV^− 1^) and the largest ∆N_max (28.0), implies a highly responsive system to external electronic perturbations such as analyte interaction. Furthermore, the electron charge transfer (ECT) values support this conclusion: ZnC59@Ac exhibits a substantial negative ECT of -4.28 eV, indicating strong electron transfer from acetone to the sensor, which is a critical mechanism for signal generation in electronic sensing.

**Fig. 5:**
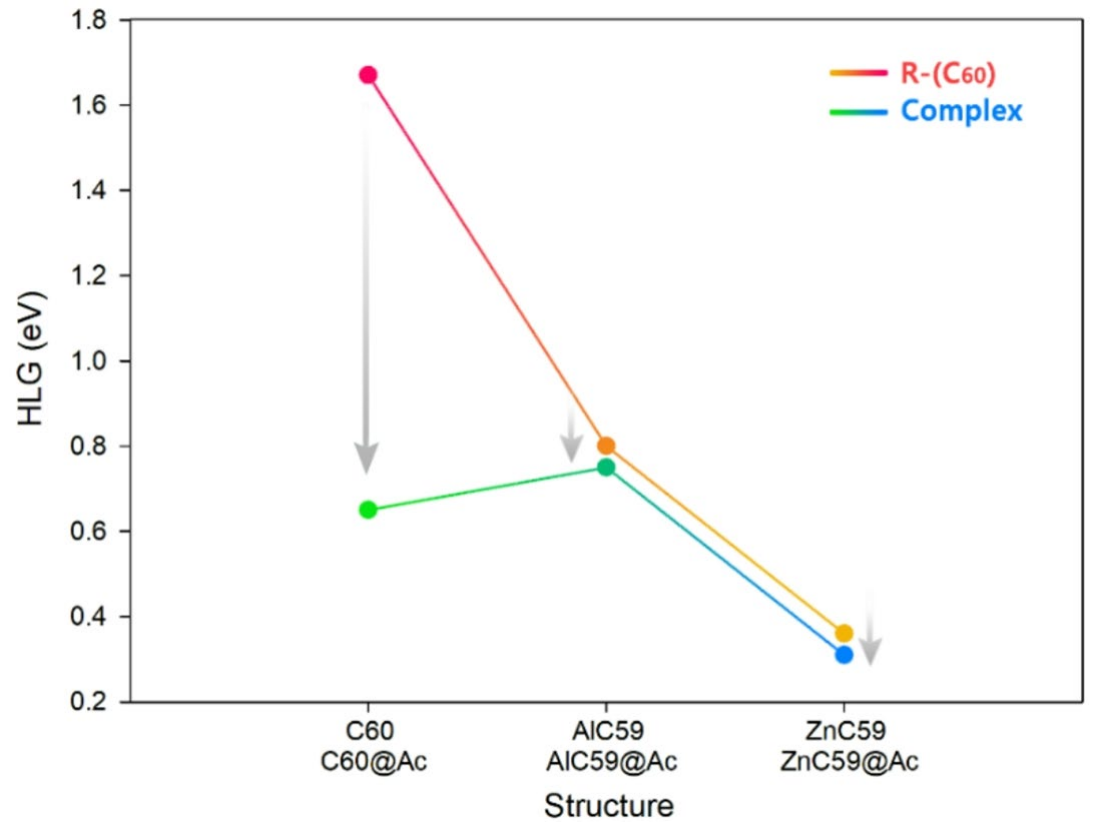
The trend of changes in the energy gap of each of the designed sensors in the presence/absence of Ac.

Figure 5 graphically illustrates the trend in energy gap variation for each structure in the presence and absence of acetone.

Finally, while all designed structures show potential for acetone detection through observable changes in electronic descriptors, ZnC_59_@Ac emerges as the most effective sensor. Its markedly low energy gap, highest softness, largest charge transfer capacity, and strong electronic interaction with acetone (evidenced by its large negative ECT) collectively indicate superior sensitivity and responsiveness. Therefore, ZnC_59_ is recommended as the most promising sensor candidate for acetone detection based on the comprehensive electronic analysis.

**Fig. 6:**
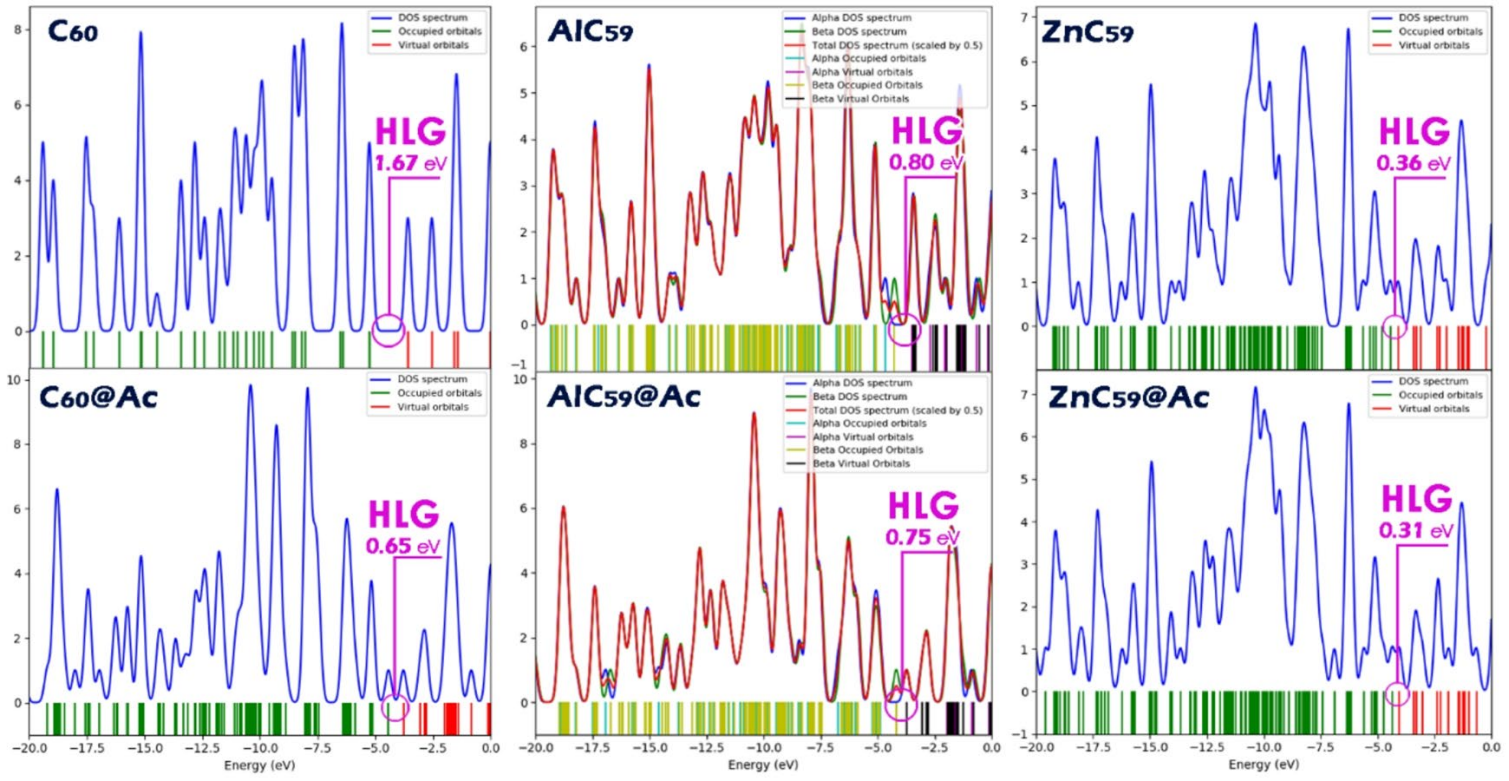
DOS plot for each of the structures studied in this work.

In this study, a Density of States (DOS) plot was employed to confirm the calculated energy gap values^43^. The DOS plot serves as a powerful visual tool for illustrating the electronic structure of molecules, particularly the distribution of electronic states and the energy gap between the HOMO and LUMO. By clearly showing the position of the HOMO and LUMO levels, the DOS diagram enables a straightforward identification of the energy gap. As shown in Fig. 6, the DOS plots for the studied systems show a stable overlap with the energy gap values reported in Table 2. This agreement between the visual representation and the numerical data confirms the reliability of the calculated energy gaps and supports the validity of the computational results.

**Fig. 7:**
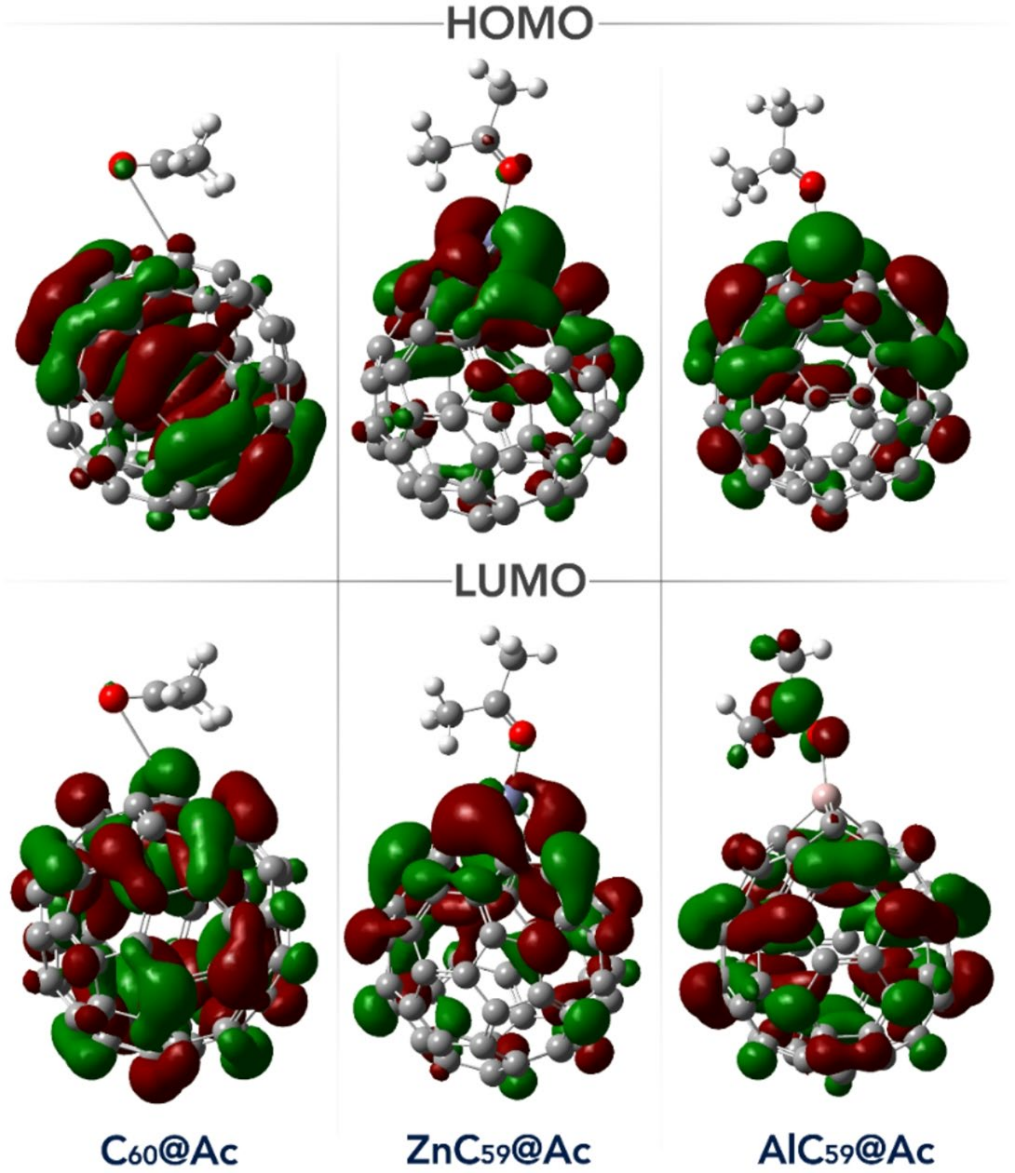
Spatial shape of HOMO/LUMO orbitals (calculated using GaussView 6.0 software (https://gaussian.com/gaussview6/)) in each of the designed complexes.

Examining the spatial shape and distribution of the HOMO and LUMO orbitals is essential for understanding a molecule’s electronic structure and predicting its chemical behavior^44^. These orbitals define the frontier regions of electron activity, where the HOMO represents the most likely site for electron donation and the LUMO corresponds to the preferred site for electron acceptance. In the context of molecular sensing, especially in donor-acceptor systems, the localization of the HOMO and LUMO determines how the sensor engages with target analytes, influencing both sensitivity and selectivity. In all the designed sensor-acetone complexes studied in this work, both the HOMO and LUMO orbitals are primarily located on the sensor structure itself (Fig. 7). This orbital localization suggests that the interaction and electron transfer occur mainly through the sensor, confirming that the sensor plays the dominant role in capturing and responding to acetone. Such a distribution is favorable for efficient sensing performance, as it ensures that the presence of the analyte directly perturbs the sensor’s electronic structure.

### Dipole moment

**Fig. 8:**
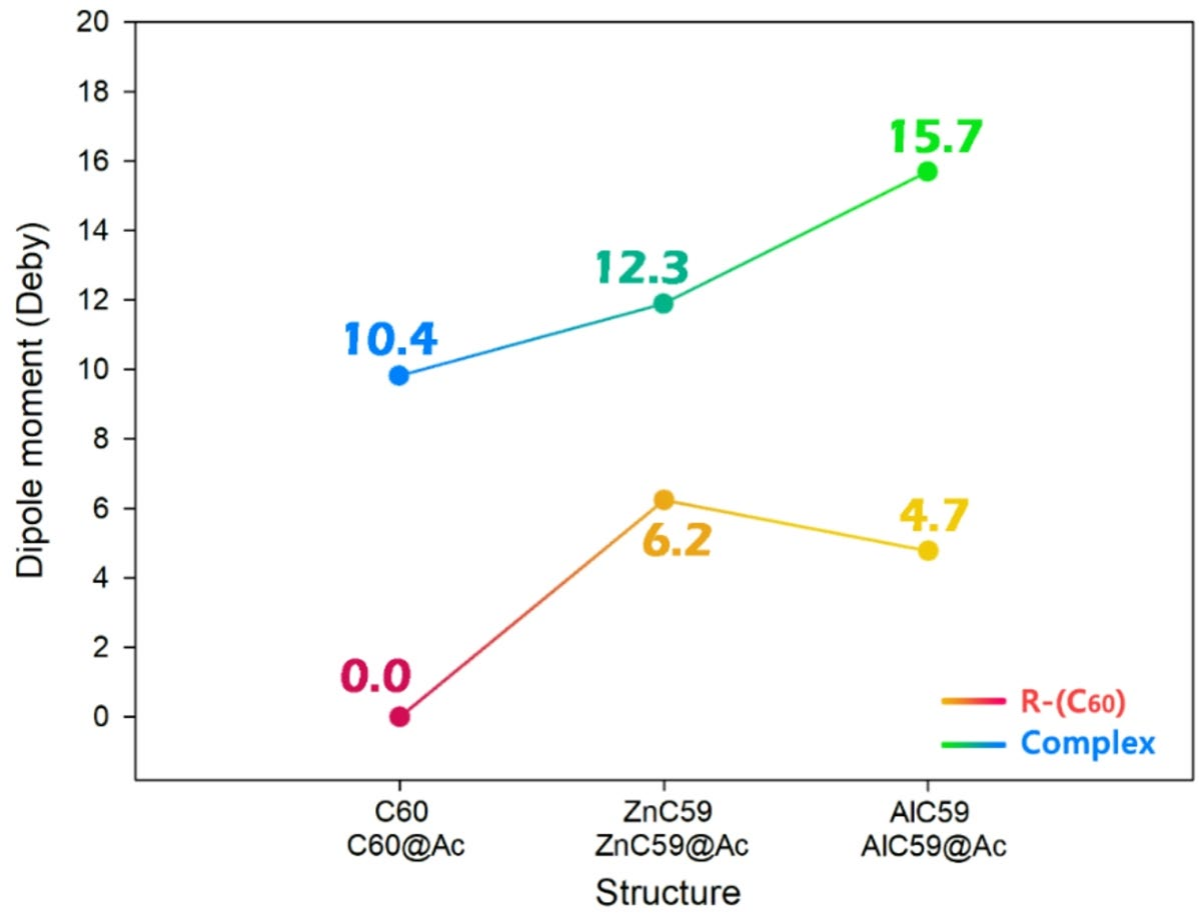
The trend of dipole moment changes in each of the designed sensors in the presence/absence of acetone.

Considering the dipole moment in sensor design is essential because it directly influences both the solubility of the sensor material and its ability to generate electrical signals upon interaction with an analyte. From the solubility perspective, a higher dipole moment generally enhances the sensor’s polarity, which can improve its compatibility with polar solvents or biological environments, thereby facilitating its dispersion and stability in solution-based applications. This is especially critical for sensors intended for use in aqueous or physiological media, where good solubility ensures consistent performance and reproducibility^45^. From the perspective of electrical signal generation, the dipole moment reflects the separation of charges within a molecule. When an analyte binds to a sensor, it can alter the electron distribution and, consequently, the dipole moment of the sensor complex. Such changes can be translated into measurable electrical signals, such as variations in conductivity, capacitance, or current, forming the basis of many electrochemical and electronic sensing mechanisms. Therefore, monitoring and optimizing the dipole moment is essential in designing sensitive and effective molecular sensors^46^. In this regard, the dipole moments of the designed sensors were calculated in the presence/absence of Ac, and the results are reported in Fig. 8.

In the absence of acetone, the pristine fullerene C_60_ exhibits a dipole moment of 0.0 Debye, indicating a highly symmetrical and non-polar structure. This lack of inherent polarity limits its ability to generate a strong electrical signal upon analyte interaction, as the system does not undergo significant electronic redistribution. In contrast, both ZnC_59_ and AlC_59_ exhibit substantial intrinsic dipole moments of 6.2 and 4.7 Debye, respectively. These elevated values result from the incorporation of metal dopants (Zn and Al), which break the molecular symmetry of the fullerene cage and induce charge polarization. This pre-existing polarity enhances the system’s sensitivity to electronic perturbations, setting the stage for more efficient signal transduction.

Upon interaction with acetone, the dipole moments of all structures increase significantly, reflecting notable electronic reorganization. For C_60_@Ac, the dipole moment rises from 0.0 to 10.4 Debye, indicating that acetone adsorption induces polarity in an otherwise symmetric system. However, the doped systems exhibit even more pronounced increases: ZnC_59_@Ac reaches 12.3 Debye, and AlC_59_@Ac achieves the highest value at 15.7 Debye. These enhancements confirm more substantial charge redistribution upon acetone binding and suggest a more effective transduction of the analyte-sensor interaction into a measurable electrical signal.

Scientifically, this mechanism of signal generation is rooted in the modulation of the sensor’s dipole moment due to molecular adsorption. As acetone binds to the sensor surface, it perturbs the electron density, leading to a shift in the center of negative and positive charges. This change in dipole moment alters the local electric field and can directly impact measurable electrochemical parameters, such as the open-circuit voltage or current flow in a device configuration.

Therefore, both ZnC_59_ and AlC_59_ demonstrate superior potential compared to pristine C_60_ in generating electrical signals upon acetone detection. Their enhanced dipole moments before and after acetone adsorption reflect a higher degree of polarizability and stronger sensor-analyte interaction. Among them, AlC_59_@Ac, with the highest dipole moment (15.7 Debye), may offer the most sensitive electrochemical response, though ZnC_59_@Ac also presents excellent potential due to its strong intrinsic and induced dipole characteristics. Overall, both doped fullerenes stand out as promising candidates for the development of effective electrochemical sensors.

### Sensor mechanism

#### Adsorption energy, recovery time, and electrical conductivity

In electrochemical sensor design, adsorption energy indicates how strongly the analyte binds to the sensor surface (moderate values ensure both effective detection and easy desorption). Recovery time refers to the speed at which the sensor returns to its initial state after detection, influencing its speed and reusability. Electrical conductivity determines how well the sensor can transmit the electronic signal upon interaction with the analyte; higher conductivity generally leads to better sensitivity. Together, these parameters are essential for optimizing sensor performance, including sensitivity, selectivity, and response time^47^. Each of these parameters was calculated, and the results were reported in Table 3.

**Table 3:** Adsorption energy (Eads), electrical conductivity (σ), and recovery time (τ) in each of the structures studied in this work.

Structure	Eads (kcal mol^− 1^)	τ (s)	σ (A m^− 2^)
C_60_	–	–	2.35 × 10^− 5^
AlC_59_	–	–	5.34 × 10^2^
ZnC_59_	–	–	2.80 × 10^6^
C_60_@Ac	-6.3	1.27 × 10^− 11^	9.90 × 10^3^
ZnC_59_@Ac	-25.1	2.51 × 10^− 8^	7.40 × 10^6^
AlC_59_@Ac	-50.2	6.30 × 10^− 4^	1.41 × 10^3^

The data in Table 3 provide critical information on adsorption energy (Eads), recovery time (τ), and electrical conductivity (σ) for pristine C_60_, its doped forms (AlC_59_ and ZnC_59_), and their complexes with acetone (Ac), allowing for a detailed analysis of their suitability as electrochemical sensors.

Starting with electrical conductivity, pristine C_60_ exhibits a very low conductivity of 2.35 × 10^− 5^ A.m^− 2^, reflecting its limited ability to transport charge carriers. Upon doping, conductivity increases dramatically, reaching 5.34 × 10^2^ A.m^− 2^ for AlC_59_ and an impressive 2.80 × 10^6^ A.m^− 2^ for ZnC_59_. This substantial enhancement in conductivity indicates that doping significantly improves the mobility of π-electrons across the fullerene framework, which is essential for generating a strong, detectable electrical signal during sensing. The higher the conductivity, the more efficiently the sensor can transduce the chemical interaction with the analyte into an electrical signal.

Adsorption energy reflects the strength of interaction between acetone and the sensor surface. Pristine C_60_ shows moderate adsorption energy (-6.3 kcal.mol^− 1^), indicating a relatively weak but detectable interaction with acetone. Upon doping, the adsorption energy increases markedly: ZnC_59_@Ac exhibits an adsorption energy of -25.1 kcal.mol^− 1^, and AlC_59_@Ac shows an even stronger adsorption of -50.2 kcal.mol^− 1^. This trend suggests that doping enhances the sensor’s affinity for acetone, potentially improving sensitivity by stabilizing the analyte on the sensor surface.

The significant variation in adsorption energies (Eads) across the studied systems, ranging from − 6.3 kcal/mol for C_60_ to -50.2 kcal/mol for AlC_59_, stems from the fundamental difference in the interaction mechanism. For pristine C_60_, the interaction with acetone is primarily physisorption, governed by weak van der Waals forces, resulting in a considerable O-C_59_ distance (3.29 Å) and a low Eads. Doping with Al or Zn introduces localized, electrophilic metal sites that strongly attract the nucleophilic oxygen atom of acetone, significantly shortening the interaction distance (to 1.89 Å and 2.01 Å, respectively) and shifting the mechanism towards chemisorption. The exceptionally high Eads for AlC_59_ is attributed to the strong Lewis acidity of the Al atom, facilitating a powerful donor-acceptor interaction, as confirmed by the high NBO stabilization energy (E^2^) (See NBO Analysis).

Recovery time is a critical practical parameter reflecting how quickly the sensor can return to baseline after analyte detection. Pristine C_60_@Ac exhibits a speedy recovery time (1.27 × 10^− 11^ s), indicating rapid desorption but possibly insufficient analyte retention for stable sensing. ZnC_59_@Ac maintains a very short recovery time (2.51 × 10^− 8^ s), balancing prompt sensor regeneration with effective analyte adsorption. In contrast, AlC_59_@Ac displays a significantly longer recovery time (6.30 × 10^− 4^ s), indicating stronger adsorption but potentially slower sensor reset, which may limit real-time or repeated use.

Combining these parameters, the mobility of π-electrons is best facilitated in ZnC_59_ and its acetone complex, as evidenced by the highest electrical conductivity values. Enhanced π-electron mobility enables efficient charge transfer and signal generation upon acetone adsorption. Although AlC_59_@Ac has the strongest adsorption energy, its comparatively low conductivity and longer recovery time may hinder rapid sensor response and signal transduction. Pristine C_60_, while exhibiting excellent recovery, lacks the electrical conductivity and adsorption strength necessary for effective sensing.

Therefore, considering the balance between strong acetone adsorption, high electrical conductivity (reflecting π-electron mobility), and reasonable recovery time, ZnC_59_ emerges as the most promising electrochemical sensor. It offers sufficient analyte affinity for detection, rapid recovery for sensor reuse, and superior electronic properties to generate a clear and responsive electrical signal. For acetone adsorption alone, AlC_59_ demonstrates the strongest binding, suggesting its potential as an efficient adsorbent, though its sensor performance may be limited by slower electron transport and recovery.

#### Natural bond orbitals (NBO)

In the design of electrochemical sensors, Natural Bond Orbital (NBO) analysis and second-order perturbation energy (E^2^) analysis play a vital role in understanding the electronic interactions between the sensor and the analyte at the orbital level. These computational tools provide insight into the nature, direction, and strength of donor-acceptor interactions that occur during the sensing process. NBO analysis allows for the decomposition of molecular wavefunctions into localized orbitals, closely resembling classical chemical bonding concepts (e.g., lone pairs, bonds, and antibonds). This enables the identification of specific orbital interactions (such as σ→σ*, LP→π*, or π•π*) that govern how electron density is redistributed when a sensor interacts with a target molecule. In the context of electrochemical sensor design and NBO analysis, π→π* transitions are considered particularly important because they often represent the dominant electronic interactions between the sensor and the analyte. These interactions are significant in electrochemical sensors, where changes in electron distribution translate into measurable electrical signals. Second-order perturbation energy (E^2^) values, derived from NBO analysis, quantify the stabilization energy associated with these donor-acceptor interactions. A higher E^2^ value indicates a stronger interaction between donor and acceptor orbitals, implying more effective charge delocalization. In the context of sensing, higher E^2^ values indicate greater charge transfer between the analyte and the sensor, which typically enhances the sensor’s sensitivity and responsiveness. These strong orbital interactions can lead to significant shifts in electronic properties (such as HOMO-LUMO gap, conductivity, and electron density distribution), which are detectable through electrochemical means^48^.

These analyses are essential tools for rational sensor design and predicting the effectiveness of a sensor in response to specific target molecules. E^2^ is calculated using Eq. 11.

11 $$\:{\varvec{E}}^{2}={\varDelta\:\varvec{E}}_{\varvec{i},\varvec{j}}-\varvec{q}\frac{{\varvec{F}}^{2}(\varvec{i},\varvec{j})}{{\varvec{E}}_{\varvec{j}}-{\varvec{E}}_{\varvec{i}}}.$$

**Table 4:** Calculated values of NBOs analysis for the studied complexes.

Complex	Donor (i)	Type	Acceptor (j)	Type	E^(2)^ kcal.mol^− 1^	E(j)-E(i) a.u.	F(i, j) a.u.
C_60_@Ac	C_1_-C_2_	$\:\sigma\:$	C_1_-C_6_	$\:{\sigma\:}^{*}$	2.61	1.09	0.048
	C_1_-C_2_	$\:\sigma\:$	C_9_-C_10_	$\:{\sigma\:}^{*}$	2.81	1.09	0.049
	C_1_-C_6_	$\:\pi\:$	C_7_-C_19_	$\:{\pi\:}^{*}$	12.73	0.25	0.051
	C_1_-C_6_	$\:\pi\:$	C_17_-C_18_	$\:{\pi\:}^{*}$	12.71	0.25	0.050
	O_61_	LP (1)	C_60_-C_62_	$\:{\pi\:}^{*}$	1.79	0.94	0.037
AlC_59_@Ac	C_1_-C_2_	$\:\sigma\:$	C_2_-C_3_	$\:{\sigma\:}^{*}$	1.27	1.08	0.047
	C_1_-C_2_	$\:\sigma\:$	C_1_-C_6_	$\:{\sigma\:}^{*}$	1.31	1.09	0.048
	C_17_-C_18_	$\:\pi\:$	C_7_-C_19_	$\:{\pi\:}^{*}$	7.30	0.25	0.054
	C_27_-C_48_	$\:\pi\:$	C_16_-C_26_	$\:{\pi\:}^{*}$	13.01	0.24	0.073
	C_35_	LP (1)	C_27_-C_48_	$\:{\pi\:}^{*}$	50.19	0.11	0.102
ZnC_59_@Ac	C_1_-C_2_	$\:\sigma\:$	C_2_-C_3_	$\:{\sigma\:}^{*}$	2.51	1.08	0.047
	C_1_-C_6_	$\:\sigma\:$	C_6_-C_17_	$\:{\sigma\:}^{*}$	2.55	1.08	0.047
	C_1_-C_6_	$\:\pi\:$	C_7_-C_19_	$\:{\pi\:}^{*}$	13.31	0.25	0.052
	C_4_-C_5_	$\:\pi\:$	C_2_-C_3_	$\:{\pi\:}^{*}$	14.32	0.24	0.053
	C37	LP (1)	C14-C15	$\:{\pi\:}^{*}$	0.52	0.24	0.010

The NBO analysis of the acetone complexes reveals distinct electronic interaction patterns that highlight ZnC_59_ as the optimal electrochemical sensor and AlC_59_ as the superior adsorbent for acetone detection. ZnC_59_@Ac demonstrates exceptional characteristics for sensing applications, primarily due to its strong π→π* interactions, particularly the C_4_-C_5_→C_2_-C_3_ transition with an E^2^ value of 14.32 kcal/mol and the C_1_-C_6_→C_7_-C_19_ transition at 13.31 kcal/mol. These robust interactions indicate efficient charge delocalization through the π-system, which is crucial for generating measurable electrical signals in electrochemical detection. The relatively high Fock matrix elements (0.052–0.053 a.u.) for these transitions further confirm good orbital overlap, facilitating electron transfer processes essential for sensor responsiveness. While its lone pair interaction (C_37_ LP→C_14_-C_15_ π*) is relatively weak (0.52 kcal/mol), the strength of its π-system interactions compensates, making ZnC_59_ particularly effective for transduction of acetone presence into electrical signals (Table 4).

AlC_9_@Ac emerges as the superior adsorbent due to its extraordinary lone pair to π* interaction (C_35_ LP→C_27_-C_48_ π*) with a remarkably high E² value of 50.19 kcal/mol, the strongest interaction observed across all complexes. This exceptionally strong donor-acceptor interaction suggests that AlC_59_ can effectively capture and retain acetone molecules through intense orbital mixing. The complex also maintains respectable π→π* interactions (13.01 kcal/mol for C_27_-C_48_→C_16_-C_26_), contributing to its overall adsorption capacity. The high Fock matrix element (0.102 a.u.) for the dominant LP→π* interaction indicates excellent orbital overlap, further supporting its superior adsorption characteristics. These properties make AlC_59_ ideal for preconcentration or removal applications where strong analyte binding is required.

The pristine C_60_@Ac complex shows weaker interactions overall, with its strongest π→π* transitions at 12.73 kcal/mol and relatively insignificant lone pair contributions (1.79 kcal/mol). While these interactions are sufficient for some sensing applications, they are substantially outperformed by both ZnC_59_ and AlC_59_ in their respective roles. The dramatic enhancement in E² values for the metal-doped complexes (particularly the 100-fold increase in lone pair stabilization for AlC_59_ compared to ZnC_59_) demonstrates the significant impact of metal incorporation on the electronic properties of these systems.

Generally, ZnC_59_’s balanced, strong π-system interactions make it ideal for electrochemical sensing where charge transfer must be both substantial and reversible, while AlC_59_’s extraordinary lone pair delocalization creates unmatched adsorption capabilities.

### NCI analysis

In the design of electrochemical sensors, Non-Covalent Interaction (NCI) analysis plays a critical role in identifying and characterizing weak intermolecular interactions between the sensor and the target analyte. These interactions, such as van der Waals forces, hydrogen bonding, and π–π stacking, are often responsible for the initial recognition and binding events that underlie sensor function. The analysis is based on plotting the reduced density gradient (RDG) against the product of the sign of the second eigenvalue of the Hessian matrix (λ_2_) and the electron density (ρ), expressed as sign(λ2).ρ. This plot distinguishes between different types of interactions: negative values of sign(λ2).ρ indicates attractive interactions (such as hydrogen bonds or π-π interactions), values close to zero correspond to weak, non-specific interactions like van der Waals forces, and positive values indicate repulsive interactions or steric hindrance. This information is particularly important in electrochemical sensor design because non-covalent interactions often dictate how well the analyte binds to the sensor surface, how stable the sensor-analyte complex is, and how efficiently charge is transferred. Understanding these weak interactions helps in optimizing sensor sensitivity, selectivity, and response time^49,50^.

**Fig. 9:**
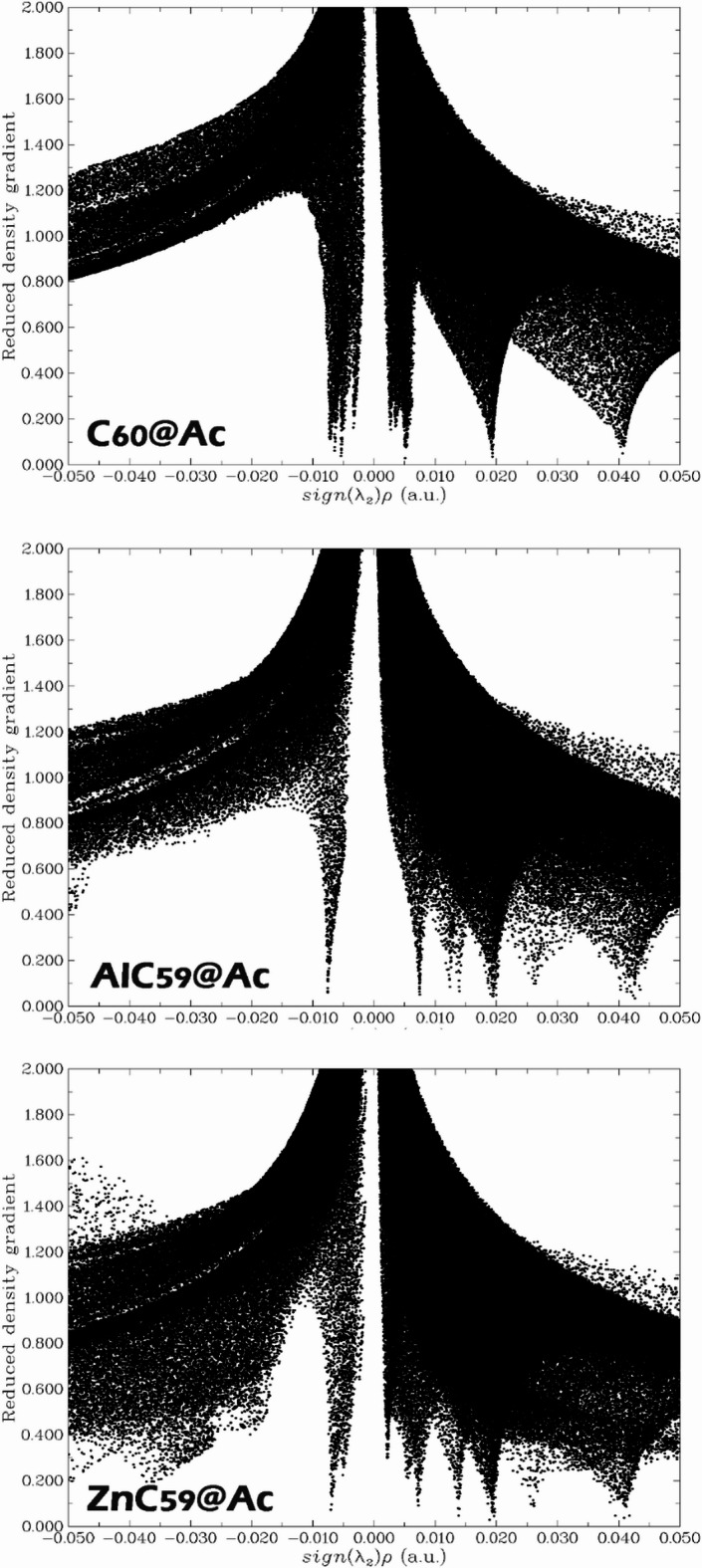
NCI contours for each of the complexes studied in this work.

The NCI (Non-Covalent Interaction) analysis for the studied complexes (C_60_@Ac, AlC_59_@Ac, and ZnC_59_@Ac) offers valuable insights into the nature, strength, and spatial characteristics of intermolecular interactions governing the binding of the Ac molecule to each fullerene-based host. By plotting the reduced density gradient (RDG) against sign(λ_2_)ρ, the NCI analysis enables a clear visualization of attractive (sign(λ_2_)ρ < 0), van der Waals (sign(λ_2_)ρ ≈ 0), and repulsive (sign(λ_2_)ρ > 0) forces [100, 101]. The comparative profiles of these complexes reveal critical differences that impact their potential utility in sensor applications (Fig. 9).

In the case of the C_60_@Ac complex, the NCI plot shows a symmetrical and relatively narrow distribution centered around sign(λ_2_)ρ ≈ 0, characteristic of dominant van der Waals interactions. The absence of significant peaks in the negative or positive regions suggests that the interaction between the neutral fullerene and Ac is mostly non-specific and weak. These findings align with the inert nature of pristine C₆₀, which interacts primarily via dispersion forces due to its closed-shell configuration and lack of strong electronic features. As a result, C_60_@Ac shows limited potential for selective or sensitive detection of Ac.

Upon doping the fullerene cage with aluminum, forming AlC_59_@Ac, a marked change is observed in the NCI contour. The plot displays a significant extension into the negative sign(λ_2_)ρ region, indicating the presence of stronger attractive interactions. This enhancement arises from the electronic modification induced by the substitution of one carbon atom with an electropositive Al atom. The resulting charge redistribution promotes stronger donor-acceptor interactions or electrostatic attractions between the AlC_59_ framework and the Ac molecule. This stronger binding is consistent with a higher calculated adsorption energy for the AlC_59_@Ac complex. However, the NCI plot for AlC_59_@Ac, while indicating stronger interactions, remains relatively symmetric and shows minimal intrusion into the positive sign (λ_2_)ρ region, suggesting that steric repulsion is not a significant factor. The overall profile reflects a balance favoring interaction strength without notable structural destabilization.

The ZnC_59_@Ac complex presents the most complex and nuanced NCI profile among the three. The contour reveals deep and broad features in the negative sign(λ_2_)ρ region, confirming the existence of significant attractive interactions, likely including electrostatic and metal–ligand-type interactions involving the zinc dopant. Notably, the plot also shows a more pronounced spread into the positive sign(λ_2_)ρ region compared to AlC_59_@Ac, indicating a moderate level of steric repulsion, possibly due to the larger atomic radius and different electronic configuration of Zn. Despite this, the richness of the attractive features and the diversity of non-covalent interactions suggest that ZnC_59_ forms a robust and specific complex with Ac. The interaction landscape includes not only dispersion forces but also strong polar interactions facilitated by the Zn site.

Although AlC_59_@Ac shows a slightly stronger interaction with Ac (as supported by both the NCI contours and by its higher adsorption energy), this does not necessarily translate into superior sensing performance. In sensor design, beyond binding strength, factors such as selectivity, reversibility, and electronic responsiveness are equally critical. ZnC_59_, despite exhibiting slightly weaker total interaction energy, offers a more favorable interaction environment for sensing due to the spatial and electronic characteristics of its NCI profile. The complex interplay of attractive and repulsive forces, combined with the enhanced polarizability and coordination capability of Zn, suggests that ZnC_59_ forms a more dynamic and responsive interface with Ac. This likely leads to more detectable changes in electronic properties (such as conductivity or charge transfer) upon binding, which are crucial for signal transduction in sensor applications.

Therefore, while AlC_59_@Ac demonstrates stronger static binding, ZnC_59_ emerges as the more effective sensing material. The NCI analysis supports this conclusion by highlighting the complex, multifaceted interactions in ZnC_59_@Ac that not only ensure stable binding of Ac but also facilitate enhanced sensor responsiveness. Consequently, ZnC_59_ is identified as the superior candidate for Ac detection, offering a balanced and sensor-optimal interaction profile.

### QTAIM

The Quantum Theory of Atoms in Molecules (QTAIM) is a widely used method for probing the nature of chemical interactions by analyzing the topology of the electron density within a molecular system. This approach focuses on critical points along the electron density gradient (specifically Bond Critical Points (BCPs)) to extract meaningful descriptors of bonding^51,52^. Key quantities such as the electron density at the BCP (ρ), its Laplacian (∇^2^(ρ(r))), and the associated energy densities (kinetic (G(r)) and potential (V(r))) are evaluated to interpret the interaction characteristics. The sum of potential and kinetic energy densities, referred to as the total energy density (Hb = V(r) + G(r)), plays a central role in categorizing the interaction. Following the classification introduced by Rozas et al., the sign and magnitude of H and ∇^2^(ρ(r)) provide insight into the interaction strength: covalent character is suggested when both are negative, intermediate interactions arise when H is positive and ∇^2^(ρ(r)) is negative, and weak, non-covalent interactions typically exhibit positive values for both^53,54^. In sensor research, these parameters help clarify whether a molecule is strongly bound to the sensor surface or only loosely associated (This information is crucial for tuning the reversibility, sensitivity, and selectivity of the sensor).

**Fig. 10:**
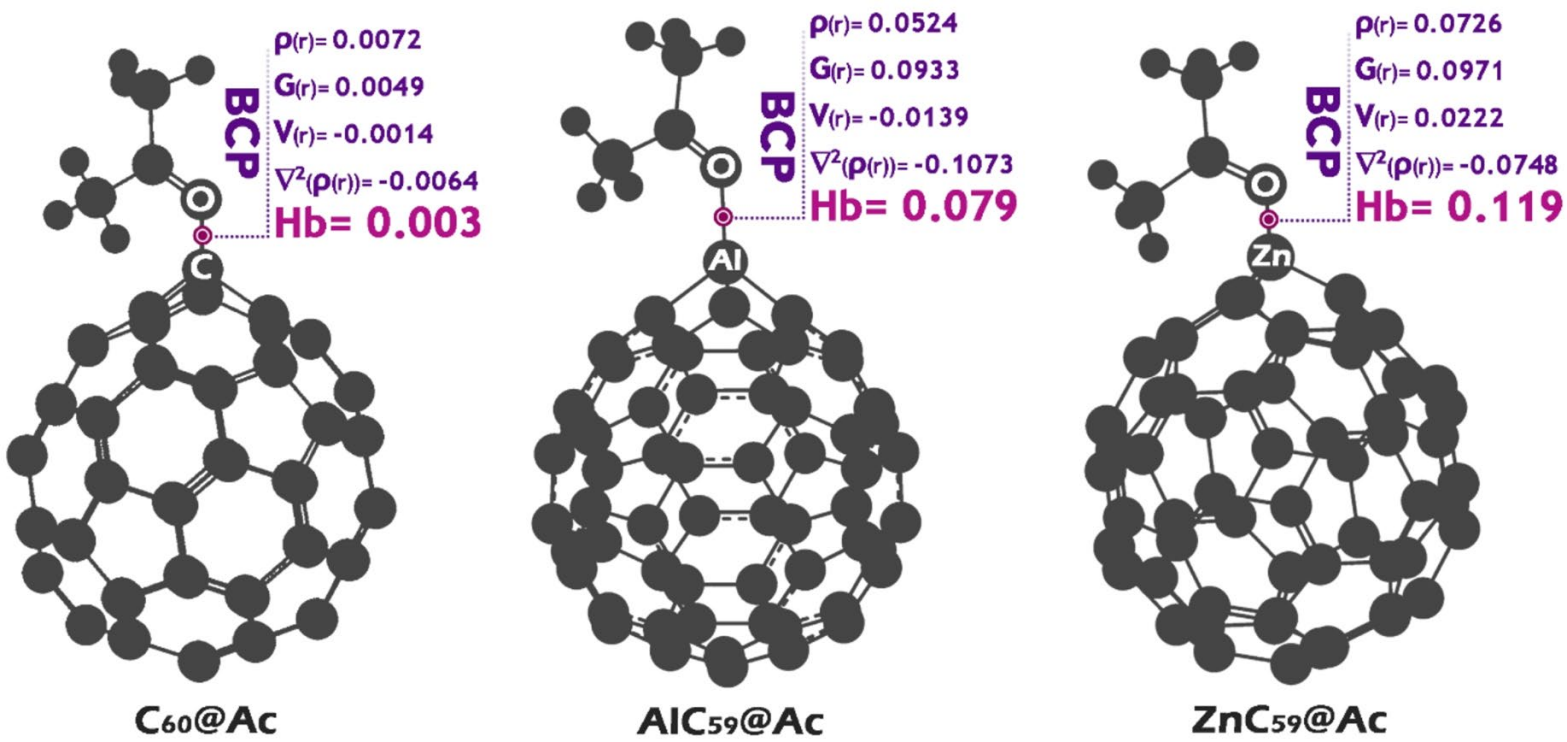
Electron density (ρ(r)), Laplacian of electron density (∇²ρ(r)), kinetic energy density (G(r)) and potential (V(r)) and total energy density (Hb) at the bond critical point (BCP) for each of the studied structures.

The data presented in Fig. 10 can be analyzed using the Quantum Theory of Atoms in Molecules (QTAIM) framework to assess the nature of chemical interactions between the sensor surface (Ac) and the respective molecules (C_60_, AlC_59_, and ZnC_59_). For C_60_@Ac, the low electron density (ρ(r) = 0.0072) and negative Laplacian (∇^2^(ρ(r)) = -0.0064) suggest a weak, non-covalent interaction. The positive total energy density (Hb = 0.003) further supports this classification, indicating that the interaction is dominated by dispersion or van der Waals forces, typical for physisorption. Such weak interactions may be reversible but lack the necessary strength for high sensor sensitivity.

In the case of AlC_59_@Ac, the higher electron density (ρ(r) = 0.0524) and strongly negative Laplacian (∇^2^ρ(r) = -0.1073) suggest a more pronounced interaction. However, the positive Hb (0.079) alongside a negative ∇^2^ρ(r) indicates an intermediate interaction, possibly with some polar or partially covalent character. While this system exhibits stronger binding than C_60_@Ac, the positive Hb suggests that the interaction is not fully covalent, which may still limit sensor stability.

The ZnC_59_@Ac system shows the highest electron density (ρ(r) = 0.0726) and a moderately negative Laplacian (∇^2^(ρ(r)) = -0.0748). Notably, both V(r) and Hb are positive (0.0222 and 0.119, respectively), indicating an intermediate-strength interaction with partial covalent character. The combination of a negative ∇^2^ρ(r) and positive Hb suggests a polar-covalent or strong donor-acceptor interaction, which is ideal for sensor applications. This balance ensures sufficient binding strength for selectivity while maintaining some reversibility for sensor regeneration. Thus, ZnC_59_@Ac emerges as the most suitable choice for sensor design, as it provides a balanced interaction (strong enough to ensure sensitivity and selectivity but not so strong as to prevent reversibility). The intermediate nature of the bonding, as evidenced by the QTAIM parameters, makes it a promising candidate for tunable sensor surfaces.

## Comparison with previous work

Compared to previous studies on fullerene-based drug adsorption and sensing, the present work demonstrates notable advantages in both adsorption capacity and sensor performance. Hosseinian et al. reported moderate adsorption energies for 5-fluorouracil on pristine C_24_ fullerenes, with limited analysis of electronic conductivity changes upon binding^55^. In contrast, our Zn-doped C_60_ (ZnC_59_) sensor exhibits a significantly lower HOMO-LUMO gap (0.31 eV) and exceptionally high conductivity (7.40 × 10^6^ A.m^-2^) after acetone binding, enabling high sensitivity and rapid signal generation. Fouegue et al. investigated pure and B-doped C_24_ nanocages for temozolomide delivery. They reported adsorption energies in the range of -15 to -30 kcal mol^-1^, whereas our Al-doped C_60_ (AlC_59_) exhibits a significantly higher adsorption energy (-50.2 kcal.mol^-1^) for acetone, making it more effective for strong capture applications^56^. Swarna et al. studied hetero-nanocages for hydroxyurea adsorption, focusing on stability and charge transfer; however, their ΔNmax values were below 10. In contrast, our ZnC59 system achieves a ΔNmax of 28.0, reflecting a far greater charge transfer capacity and electronic responsiveness^57^. Mahani et al. examined C_24_ for pyridine derivatives and found recovery times in the millisecond range, whereas ZnC59 in this work recovers in only 2.51 × 10^− 8^ s, enabling ultrafast sensor regeneration^58^. Finally, Baei and Shojaei evaluated toluene adsorption on C_24_ and doped fullerenes, reporting modest conductivity changes upon adsorption. In contrast, our Zn-doped C_60_ not only provides strong adsorption but also maintains the highest conductivity among all studied systems, ensuring superior signal transduction^59^. Collectively, this work surpasses prior studies in adsorption strength, charge transfer, electronic conductivity, and recovery time, with ZnC_59_ emerging as a high-performance, reusable electrochemical sensor and AlC_59_ as an exceptional adsorbent for environmental acetone removal.

## Conclusion

This study presents a comprehensive theoretical investigation into the adsorption and detection of acetone (Ac) using pristine and metal-doped fullerene C_60_ structures (C_60_, AlC_59_, and ZnC_59_), with the dual objective of advancing non-invasive diagnostic tools for type 2 diabetes and developing efficient adsorbent materials for environmental applications. The performance of each designed system was assessed through a multi-faceted analysis that included adsorption energy, electronic and quantum chemical descriptors, electrostatic potential mapping, natural bond orbital (NBO) analysis, non-covalent interaction (NCI) contours, and quantum theory of atoms in molecules (QTAIM).

Numerical results show clear differences in behavior between the pristine and doped fullerenes. Adsorption energies revealed a significant enhancement in binding strength upon doping: while C_60_@Ac exhibited a modest adsorption energy of -6.3 kcal/mol, the values for ZnC_59_@Ac and AlC_59_@Ac were − 25.1 kcal/mol and − 50.2 kcal/mol, respectively. This confirms that both dopants significantly improve acetone adsorption, with AlC_59_ showing the strongest affinity. However, adsorption strength alone does not determine sensor efficiency. When other key metrics are considered (such as recovery time, electrical conductivity, dipole moment change, and charge transfer capacity), ZnC_59_ outperforms its counterparts. Specifically, ZnC_59_@Ac exhibits a high conductivity of 7.40 × 10^6^ A.m^− 2^ and a short recovery time of 2.51 × 10^− 8^ s, indicating excellent responsiveness and recyclability, which are essential for real-time sensor applications.

Electronic properties further support the superior sensing performance of ZnC_59_. Its acetone complex showed the lowest HOMO-LUMO gap (0.31 eV), the highest softness (3.22 eV^− 1^), the largest charge transfer value (∆Nmax = 28.0), and a significant electrophilicity-based charge transfer (ECT = -4.28 eV), all indicative of strong and dynamic electronic interaction between the sensor and acetone. These properties make ZnC_59_ highly sensitive to electronic perturbations upon acetone binding, ensuring a measurable and reliable signal output. Additionally, dipole moment analysis demonstrated that ZnC_59_@Ac reaches 12.3 Debye, signifying substantial electronic redistribution and further enhancing signal generation capability. Although AlC_59_@Ac exhibits the highest adsorption energy and a remarkable dipole moment (15.7 Debye), which favors strong retention and signal sensitivity, it suffers from lower conductivity (1.41 × 10^3^ A.m^− 2^) and a longer recovery time (6.30 × 10^− 4^ s), potentially limiting its application in rapid and reversible sensing systems. These attributes, however, make AlC_59_ highly suitable as an efficient adsorbent material for environmental remediation, where strong, irreversible capture of volatile organic compounds like acetone is desirable.

NCI contour analysis confirmed the nature of the interactions, showing more extensive and deeper attractive regions in AlC_59_@Ac and ZnC_59_@Ac compared to the weaker, van der Waals-dominated C_60_@Ac. Notably, ZnC_59_@Ac demonstrated a more balanced interaction profile, combining strong attraction with manageable steric repulsion, indicating a stable yet reversible binding (a key feature in sensor design). Supporting this, QTAIM analysis revealed that ZnC_59_@Ac possesses the highest electron density at the bond critical point and a moderately negative Laplacian, suggesting intermediate-strength, polar-covalent interactions that balance sensitivity with reversibility. In contrast, AlC_59_@Ac showed signs of stronger, more covalent-like interaction, in line with its role as an adsorbent.

In summary, this work identifies ZnC_59_ as a highly effective electrochemical sensor for acetone detection, offering optimal performance in terms of sensitivity, selectivity, and reusability, making it an excellent candidate for non-invasive diabetes diagnostics via breath acetone monitoring. Conversely, AlC_59_, with its superior adsorption energy and stable binding characteristics, emerges as a promising material for environmental applications aimed at acetone capture and removal. The dual applicability of these materials highlights the versatility of doped fullerenes and paves the way for future developments. To build upon these findings, subsequent research should focus on the experimental synthesis and validation of ZnC_59_ and AlC_59_ complexes to confirm the theoretical predictions. Future directions include integrating ZnC_59_ into flexible, wearable platforms for real-time breath monitoring; exploring alternative dopants (e.g., Fe, Cu, B) to optimize selectivity for a broader range of volatile organic compounds; and developing hybrid materials by combining these doped fullerenes with biocompatible polymers or metal-organic frameworks to enhance stability and environmental resilience. Long-term studies on biocompatibility and sensor repeatability under physiological conditions, as well as the development of multifunctional platforms capable of both sensing and adsorbing harmful VOCs, will be crucial for translating these theoretical insights into practical healthcare and environmental technologies.

## Data Availability

The datasets used and/or analysed during the current study available from the corresponding author on reasonable request.
